# Case report of gastric outlet obstruction from metastatic lobular breast carcinoma

**DOI:** 10.1186/s12876-015-0350-y

**Published:** 2015-09-25

**Authors:** Alexander H. Kim, M. Joshua Shellenberger, Zong Ming Chen, Jinhong Li

**Affiliations:** Geisinger Medical Center, 100 N. Academy Ave., Danville, PA 17822 USA

**Keywords:** Gastric outlet obstruction, Breast cancer, Cancer, Abdominal pain

## Abstract

**Background:**

The most common malignancy to cause gastric outlet obstruction is primary gastric adenocarcinoma and it is followed by carcinoma of the pancreas and gallbladder. Herein, we report a case of gastric outlet obstruction secondary to metastatic lobular breast carcinoma.

**Case presentation:**

Fifty-seven year old Caucasian female with recently diagnosed metastatic lobular breast carcinoma to skin was referred to gastroenterology for evaluation of dyspepsia and dysphagia. She has past medical history significant for acid reflux and Clostridium difficile colitis. Computed tomography of her abdomen showed diffused bowel wall thickening without evidence of bowel obstruction. Due to persistent abdominal pain, an upper endoscopy was performed. The upper endoscopy showed gastritis and gastric stenosis in the gastric antrum. These lesions were biopsied and dilated with a balloon dilator. The biopsy of the gastric antrum later showed a metastatic carcinoma of breast origin with typical tumor morphology and immune-phenotype.

**Conclusions:**

Differentiating metastatic breast carcinoma from primary gastric adenocarcinoma cannot be done using histological examination alone. Immunohistochemistry is needed to differentiate the two based on staining for estrogen and progesterone receptors. The presence of gross cystic disease fluid protein 15 is also suggestive of metastatic breast carcinoma. The stomach has a significant capacity to distend (up to 2–4 L of food) and malignant gastric outlet obstruction is often undetected clinically until a high-grade obstruction develops. Our case demonstrates valuable teaching point in terms of broadening our differentials for gastric outlet obstruction. When patients present with gastric outlet obstruction, both non-malignant and malignant causes of gastric outlet obstruction should be considered. Once adenocarcinoma has been determined to be the cause of gastric outlet obstruction, further immunohistochemistry is needed to differentiate breast carcinoma from other carcinomas.

## Background

Gastric outlet obstruction (GOO) can be a complication of malignancy, gastric polyps, peptic ulcer disease, or gallstone obstruction. Since the advent of histamine-2 blocker in the late 1970s, the incidence of GOO from duodenal ulcer has declined dramatically and the malignancy accounts for as many as 61 % of GOO [1]. The most common malignancy to cause GOO is primary gastric adenocarcinoma and it is followed by carcinoma of the pancreas and gallbladder [1, 2]. Herein, we report a case of gastric outlet obstruction secondary to metastatic lobular breast carcinoma. There are several case reports regarding GOO secondary to metastatic breast cancer and it is an extremely rare cause of obstructions per literature review from 1995 to date.

**Fig. 1:**
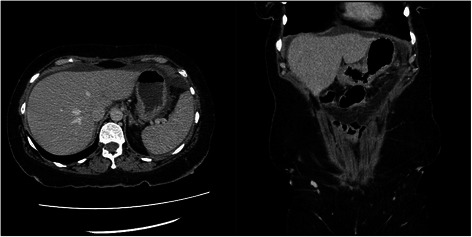
EGD. Esophagogastroduodenoscopy images showing gastric outlet obstruction at the gastric antrum

**Fig. 2:**
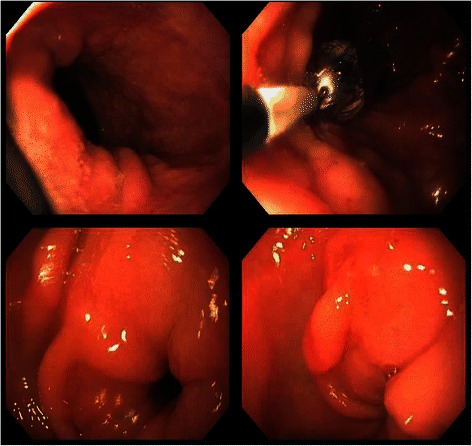
CT scan. CT scan of abdomen without IV or PO contrast showing thickening of the gastric antrum

**Fig. 3:**
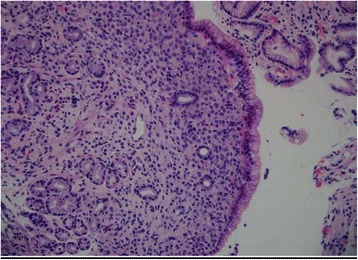
Lower power field. Lower power shows relatively bland looking tumor cells expanding the lamina propria of the mucosa

**Fig. 4:**
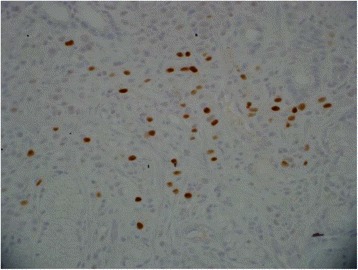
Estrogen receptor. The tumor cells are positive for nuclear staining of estrogen receptor

**Fig. 5:**
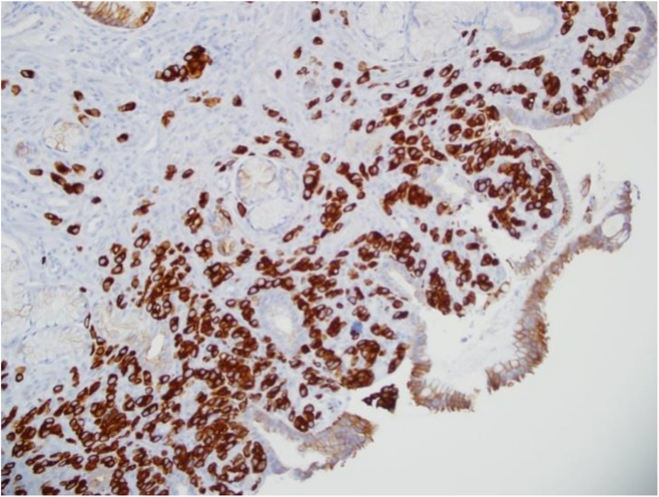
Cytokeratin 7. The tumor cells in the lamina propria are positive for cytokeratin 7 immunostaining

**Fig. 6:**
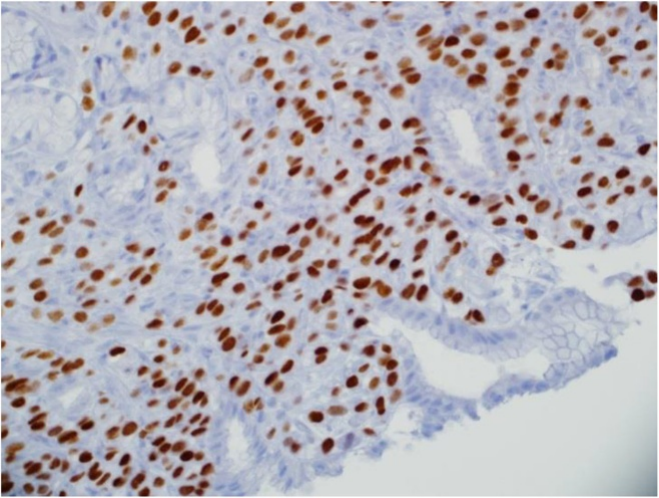
GATA-3. The tumor cells show positive nuclear stain for GATA-3 (a marker for breast cancer)

## Case presentation

Fifty-seven year old female with recently diagnosed metastatic lobular breast carcinoma to skin was referred to gastroenterology team for evaluation of dyspepsia and dysphagia. She has past medical history significant for gastroesophageal reflux disease (GERD) and Clostridium difficile colitis. She was previously evaluated in the emergency department for abdominal pain. CT scan of her abdomen showed diffused bowel wall thickening without evidence of bowel obstruction (Fig 1). Her CBC was unremarkable without leukocytosis. LFT’s were not elevated and lipase level was normal at 30 U/L. Due to absence of acute medical issue, she was discharged to home from the emergency department. The patient was seen by oncology team as outpatient soon after her recent ED visit for further evaluation of the recently diagnosed breast cancer by skin biopsy under the right breast. The biopsy showed a metastatic lobular carcinoma and the estrogen receptor on these cells was strongly positive in 100 % of the cells. The progesterone receptor was negative and the Her-2/neu expression was negative by the FISH assay. She continued to complain of abdominal pain and difficulty with swallowing to her oncologist. An esophagogastroduodenoscopy (EGD) was performed subsequently to evaluate her dyspepsia and dysphagia. The EGD showed gastritis and gastric stenosis in the gastric antrum (Fig 2). These lesions were biopsied and dilated with a balloon dilator. The biopsy of the gastric antrum later showed a tumor with morphology of relatively uniform cells growing in single files in the lamina propria (Fig 3). The immune-reactivity was positive for cytokeratin 7, gross cystic disease fluid protein (GCDFP) 15, estrogen receptor (ER) and GATA-3 (Figs 4, 5 and 6). The immunostaining profile and tumor morphology were consistent with a metastatic carcinoma of breast origin.

## Conclusions

Differentiating metastatic breast carcinoma from primary gastric adenocarcinoma cannot be safely done using histological examination alone. However, less pleomorphic tumor morphology and single file pattern are commonly seen in lobular carcinoma of breast. Immunohistochemistry is required to differentiate the two based on staining for a panel of immnohistochemical markers including GATA-3, CDX-2, GCDFP-15, and estrogen receptors. While GATA-3 is currently considered as a better marker for breast cancer, diffuse strong ER positivity and presence of GCDFP 15 are also suggestive of metastatic breast carcinoma [3]. GATA3 is a sensitive and specific marker for diagnosis of breast carcinomas [4]. The stomach has a significant capacity to distend (up to 2–4 L of food) and malignant GOO is often undetected clinically until a high-grade obstruction develops [5]. Gastrointestinal (GI) metastasis from breast cancer is extremely rare and accounts for less than one percentage of metastatic breast cancers [6]. The most common sites of GI track metastasis from breast cancer are colon and rectum [7]. Metastasis to stomach only accounts for a small fraction of GI track metastasis from breast cancer [8]. Gastrointestinal metastasis from breast is associated with poor prognosis [9]. Our case demonstrates valuable teaching point in terms of broadening our differentials for GOO. When patients present with GOO, both non-malignant and malignant causes of GOO should be considered. Once adenocarcinoma has been determined to be the cause of GOO, further immunohistochemistry is needed to differentiate breast carcinoma from other carcinomas.

## Consent

Written informed consent was obtained from the patient for publication of this Case report and any accompanying images. A copy of the written consent is available for review by the Editor of this journal.

## Abbreviations

GOO: Gastric outlet obstruction
GERD: Gastroesophageal reflux disease
CT: Computed tomography
CBC: Complete blood count
LFT: Liver function test
ED: Emergency department
FISH: Fluorescence in situ hybridization
EGD: Esophagogastroduodenoscopy
ER: Estrogen receptor
GCDFP 15: Gross cystic disease fluid protein 15
